# Membranous Ophthalmia

**Published:** 1891-03-14

**Authors:** 


### OPHTHALMOLOGY.

### MEMBRANOUS OPHTHALMIA.

In some text books the term Diphtheritic Ophthalmia is given to all cases of purulent ophthalmia in which there is a membranous exudation on the conjunctiva. As a matter of fact true diphtheretic ophthalmia is very uncommon, at any rate in England. Membranous ophthalmia, as ordinarily met with, is only a variety of purulent ophthalmia; but it is a variety which clinically much resembles true diphtheria of the conjunctiva and demands somewhat similar treatment. Still, the distinction is important, and in the rare cases in which diphtheria primarily attacks the conjunctiva the diagnosis is very difficult. Investigations which have been conducted for the past two years at the Pasteur Institute by M.M. Roux and Yersin have proved that in all cases diphtheria depends on the bacillus of Loeffler which they have found in large numbers in the necrotic membrane formed. Cultivations of these bacilli are characteristic in their growth; and inoculations of them in guinea-pigs cause nearly all the symptoms of diphtheria, including in some cases diphtheritic paralysis. Unfortunately, this test, though conclusive if properly used, is not very easily applied, and may fail even in the hands of skilled experimenters. Prudden (*American Journal of Medical Science*, 1889), after careful observations in a number of cases thought to be undoubtedly diphtheria, could find no bacilli, and the results he obtained were mainly negative. So that, although these discoveries are of great scientific value, other means must be taken to distinguish between membranous and diphtheritic ophthalmia. There are in most cases the following differences: In diphtheritic ophthalmia the injection of the conjunctiva is of a dusky red colour, there is less discharge, and from the outset it is thin and watery; patches of membrane form early, and are very adherent to the conjunctiva, and when removed bleeding results; and, lastly, the general health of the patient is much more affected. Ordinary membranous ophthalmia generally begins like gonorrhœal ophthalmia, but owing to some cause the previous creamy and profuse discharge of pus ceases, membranous exudation appears on the conjunctiva, and the simple inflammatory œdema of the lids is replaced by a solid exudation rendering them almost rigid. It occurs usually as a result of purulent inoculation in those who are in bad health, such as ill-fed children, and women recovering from confinement; or it may be due to the excessive irritation produced by the too frequent application of caustics to the conjunctiva. The chief danger in both classes of cases is that the swelling of the conjunctiva may cause necrosis and sloughing of the cornea by interfering with its nutrition. To avoid this, the ordinary remedies suggested in text-books are usually effectual; but there are one or two points in which we are inclined to differ from what may be termed the orthodox routine treatment. First, in membranous ophthalmia (but never in diphtheritic), if the case is seen early, before the membrane is thick and the lids brawny with solid œdema, we advise a free application of a solution of nitrate of silver (10 grains to the ounce) to the everted palpebral conjunctiva. We have seen cases in which the attack has certainly been cut short by this treatment, but its effect ought carefully to be watched, and if the result is good it may be repeated in two days. If the lids become at all more brawny, and the skin more tense and glazed, it should not be repeated. Perchloride of mercury lotion (1-5,000) is generally the best, but if it causes pain and the lids are very rigid and swollen, and there is much chemosis of the conjunctiva, then undoubtedly the best application is sulphate of quinine lotion (grs. iv. to the ounce, dissolved in a minimum of sulphuric acid) as suggested by Mr. Tweedy. The lids are separated, "the conjunctival sac being converted, as it were, into a trough holding quinine lotion," then small pads of cotton wool thoroughly wetted with the lotion are kept loosely applied and frequently changed. The lotion should be applied at least six or eight times a day; it is a good antiseptic, and hardly at all irritating to the conjunctiva. Generally speaking, cold or iced lotions are the most comforting and most suitable, but if there is still much pain, and after fair trial the swelling of the conjunctiva does not subside, frequent hot fomentations should be tried (saturated solution of boric acid or hot quinine lotion are best), and will often answer better than cold applications. In cases where the cornea becomes hazy and sloughing is threatened, owing to the extreme swelling of the ocular conjunctiva, Hansen Grut, of Copenhagen, has excised a ring of conjunctiva all round the cornea. We have had no experience of this treatment, but should prefer to have recourse to the ordinary method of scarifying the conjunctiva by making a number of radial incisions. Some have attempted the removal of the membrane in diphtheritic ophthalmia by the aid of citric or lactic acid, but this would seem likely to cause irritation and be harmful. Extreme care must be exercised not to injure the cornea when applying remedies: an abrasion will most likely lead to an ulcer. Finally, all cases of membranous or diphtheritic ophthalmia need tonics and stimulating diet, and when the general health will permit, fresh air and exercise. The heroic treatment by bleeding from the arm (even three or four times, and each time until fainting was caused), and violent purgation, that seems to have been the recognised plan at the beginning of this century for purulent ophthalmia of all kinds, has proved to be not only ineffectual but harmful. In fact, bleeding, or lowering treatment of any kind, is hardly ever desirable in gonorrhœal ophthalmia, and still less so in the membranous or diphtheritic varieties.

